# Controlled Glycolysis of Poly(ethylene terephthalate) to Oligomers under Microwave Irradiation Using Antimony(III) Oxide

**DOI:** 10.1021/acsapm.3c01071

**Published:** 2023-07-12

**Authors:** Somayeh Mohammadi, Martin G. Bouldo, Mojtaba Enayati

**Affiliations:** Center for Materials and Manufacturing Sciences, Departments of Chemistry and Physics, Troy University, Troy, Alabama 36082, United States

**Keywords:** microwave irradiation, PET glycolysis, chemical recycling, PET oligomers, catalytic depolymerization

## Abstract

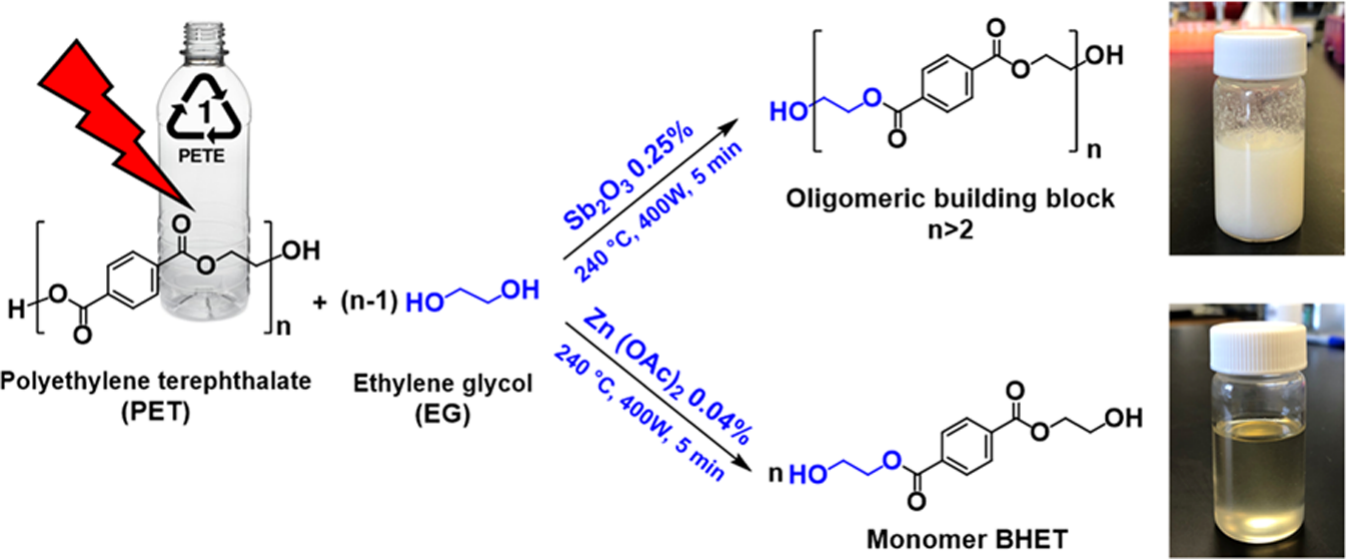

We report here the production of higher-order oligomers from the glycolysis of poly(ethylene terephthalate) (PET) by using microwave irradiation in a controlled fashion, instead of its fully glycolyzed product, bis(2-hydroxyethyl)terephthalate (BHET). We show that different catalysts can generate either BHET as the ultimate glycolysis product or higher oligomers of PET under microwave irradiation. Depolymerization of waste PET with an average degree of polymerization (DP) of 417 from water bottles was performed in the presence of 0.25 wt % antimony(III) oxide (Sb_2_O_3_) as the catalyst at 240 °C and 400 W microwave power, resulting in an oligomer yield of 96.7% with an average DP of 37. Under these conditions, the conversion of PET to oligomers reached 100% in only 5 min at 240 °C (with a 10 min ramping time) and with a ethylene glycol to PET weight ratio of 2.5. In comparison, under the same reaction conditions, 0.04 wt % of zinc acetate (Zn(OAc)_2_), a well-known catalyst for PET glycolysis, produces only the BHET monomer in 96.3% yield. Our results demonstrated that by using Sb_2_O_3_, the same catalyst that is used extensively for PET synthesis from BHET, under microwave irradiation, the PET glycolysis can be controlled to produce higher PET oligomers as an alternative for a complete chemical depolymerization to the BHET monomer. These oligomers are more suitable for being used as additives for many applications and to produce high-quality second-generation products, including regenerated PET.

## 1 Introduction

In recent years, the increasing use of polymers, specifically poly(ethylene terephthalate) (PET), in various industries and applications has resulted in the production of a significant amount of solid waste in the world.^1^ Considering the environmental pollution problems caused by PET waste, many research groups are trying to develop reliable and convenient recycling methods for reusing PET waste in order to bring it back into the material cycle.^2,3^ Three methods have been developed for the recycling of PET waste, including mechanical, thermal, and chemical processes.^3,4^ Most of the popular mechanical recycling methods consist of collecting, sorting, crushing, washing, and melt-granulating the plastic waste, then re-introducing it into the production cycle. However, although mechanical recycling has its advantages, due to thermal degradation, the regenerated polymers produced are of lower quality when compared to the first generation (downcycling). One common approach for producing high-quality recycling products and preventing downcycling is the chemical depolymerization of waste polymers to produce second-generation monomers. However, chemical depolymerization still has several drawbacks such as usage of excess solvents, toxicity from catalysts, difficulty in monomer purification, high energy consumption, and financial constraints, making this method undesirable for industrial polymer recycling.^5−7^

A reasonable alternative for complete chemical depolymerization to a monomer would be controlling the depolymerization reaction to produce specific oligomeric building blocks by partial depolymerization. Oligomer building blocks are suitable for high-quality second-generation products that can be produced via re-polymerization or copolymerization methods.^8^ Due to their low solubility, isolation and purification of oligomers is easier compared to monomers, and can be achieved with conventional engineering process methods. The process of oligomer synthesis also consumes less energy. In recent years, some research groups have used the hydroxyl terminated oligomers synthesized by glycolysis of PET as a starting material for synthesizing polymers like polyurethanes,^9^ unsaturated polyesters,^10^ and UV curable films.^11^

Different types of chemical depolymerization, including methanolysis,^12^ hydrolysis,^13^ ammonolysis,^14,15^ and glycolysis,^16^ have been used for PET depolymerization. The glycolysis approach is one of the processes that have become more attractive for increasing the efficiency and decreasing the industrial costs of the reaction by using various catalytic systems,^17^ supercritical conditions,^18,19^ and microwave irradiation.^20^ Among these techniques, the use of microwave radiation has garnered significant attention as an energy source for these reactions. Unlike the conventional heating process, microwave radiation can offer a fast heating rate, which makes it an ideal choice for reducing the reaction time. Also, it is possible for the reaction to follow a different path using microwave irradiation compared to conventional heating.^21^

Depolymerization of PET under microwave irradiation has been studied in the presence of different nucleophiles such as hydrazine,^22^ ammonia,^23^ alkali hydroxide,^24^ propylene glycol,^25^ ethylene glycol (EG),^26^ and ethanolamine^27^ to produce various chemical products. All of these studies focus on PET glycolysis to produce bis(2-hydroxyethyl)terephthalate (BHET) and other related monomeric units as the main product, i.e., ultimate depolymerization, without considering the fact that with mild reaction conditions, oligomers of different molecular weights can be produced. In 2013, Roy’s group^20^ described the use of zinc acetate (Zn(OAc)_2_) as a catalyst under microwave conditions for the preparation of polyester polyols from PET by reacting it with diols of different molecular weights. The resulting polyols were reacted with isocyanate in the presence of a surfactant, blowing agent, and catalyst to prepare polyurethane foams. At the same time, Kandelbauer’s group^28^ synthesized oligomeric ethylene terephthalate with defined degrees of polymerization by melt-mixing PET with different quantities of adipic acid.

In our previous study, we showed that the antimony(III) oxide, the most industrially used catalyst for PET synthesis, can efficiently depolymerize PET to BHET in a glycolysis reaction in only 1 h at 200 °C and 2.1 bar.^29^ In the present study, depolymerization of waste PET from drinking water bottles via glycolysis under microwave irradiation has been studied with the aim of producing oligomeric building blocks of PET instead of producing the ultimate degradation product, BHET. Our results showed that the post-consumer PET can be efficiently converted to the PET oligomeric building blocks (and not the BHET) in 96.7% yield in the presence of 0.25 wt % antimony(III) oxide (Sb_2_O_3_) at 240 °C for 5 min (with a 10 min ramping time), while in the same conditions, Zn(OAc)_2_ produces only BHET and no higher oligomers. The effect of catalyst loading, EG/PET weight ratio, reaction time, and reaction temperature were studied and the results were compared with the well-known catalyst for PET glycolysis, zinc acetate (Zn(OAc)_2_). Our results demonstrated that zinc acetate produces BHET in high yield (96.3%) under microwave irradiation not only with the lowest level of catalyst (0.04% w/w) but also in the shortest reaction time (10 min ramping time and 5 min holding time, power 400 W, temperature 240 °C). By increasing the Zn(OAc)_2_ loading to 0.25 wt % and reaction time to 25 min, the yield of oligomeric building blocks’ production became 45.2%, which means the efficiency of Zn(OAc)_2_ for BHET production was decreased.

## 2 Materials and Methods

### 2.1 Materials

Commonly used 500 mL PET water bottles were acquired from the local market for this study. After removing the bottles’ labels and the cap, an industrial shredder (Brabender CWB, Granu-Grinder M120/150) was used to cut them into 2–5 mm flakes. The shredded PET flakes were washed with methanol and vacuum-dried at 60 °C. Commercial BHET was purchased from Sigma-Aldrich and used as our standard. Antimony(III) oxide (Sb_2_O_3_) was supplied from Acros Organic Co. and zinc acetate (Zn(OAc)_2_) was purchased from Alfa Aesar. Ethylene glycol (EG) and methanol were acquired from Fisher Chemical. All chemical reagents were used without further purification.

### 2.2 Characterization

Fourier transform infrared (FTIR), nuclear magnetic resonance (NMR), thermogravimetric analysis (TGA), differential scanning calorimeter (DSC), high-pressure liquid chromatography (HPLC), and viscometer analysis were used for characterization of the products. A Perkin-Elmer (Waltham, MA) FTIR spectrophotometer was used in the wavenumber range of 400–4000 cm^–1^ with a resolution of 4 cm^–1^ and 64 scans to identify the main functional groups. ^13^C NMR and ^1^H NMR spectra were acquired by an Ascend TM (Bruker, Switzerland) spectrometer (400 MHz) in order to investigate the glycolyzed product’s structure. DSC-250 (TA Instruments, Delaware) using Tzero Pans was used to measure thermal transitions with a heating rate of 10 °C min^–1^ from 40 to 300 °C. Thermal stability was studied using TGA-550 (TA Instruments, Delaware) with platinum-HT sample pans with a heating rate of 20 °C min^–1^ from room temperature to 800 °C. In order to identify the qualitative composition of the main products and the quantitative BHET yield, HPLC analysis (HPLC, Shimadzu, LC-10AT, UV–Vis detector) was performed with a Poroshell 120 column (EC-C18, 2.7 μm, 3.0 × 150 mm^2^) and a mobile phase of methanol and H_2_O (75–25, v/v), column temperature of 25 °C, flow rate of 0.2 mL min^–1^, injection volume of 5 mL, and UV detector set up at 254 nm. Five BHET (Sigma-Aldrich) solutions with concentrations of 250, 500, 750, 1000, and 1500 ppm were prepared in methanol. These solutions were used to prepare a calibration curve for HPLC measurement by graphing the BHET peak area at 4.41 min against the concentration of BHET in the standard solution. HPLC measurements were performed in triplicate. In order to measure the molecular weight of the PET waste and its oligomeric products, dilute-solution viscometry was used. A full description of the method is provided in Section 3.5.

### 2.3 Microwave-Assisted Glycolysis of PET

**Scheme 1:**
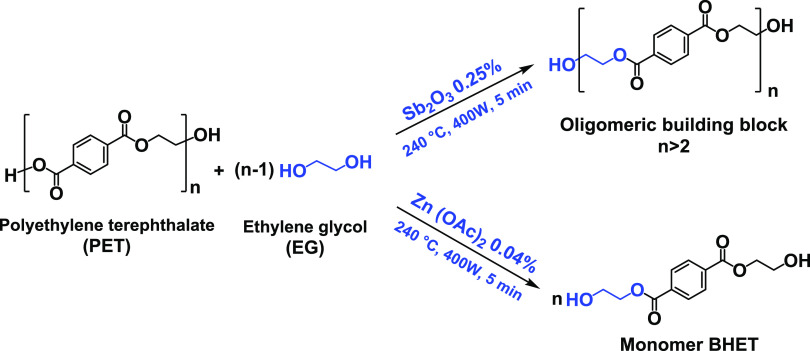
Microwave-Assisted Glycolysis of PET with EG in the Presence of Antimony(III) Oxide and Zinc Acetate as the Catalyst

A laboratory microwave oven (Mars 6, CEM Corp.) with a magnetron source for microwave generation (2.45 GHz, maximum power: 1600 W) was used for the glycolysis. Glycolysis reactions were done in an EasyPrep closed Teflon vessel, which was placed in the microwave reactor equipped with a thermometer and a magnetic stirrer. In all runs, 6.0 g of shredded PET waste was mixed with EG. The EG/PET weight ratio in different runs varied between 1.5 and 5.0 in both the presence and absence of the catalyst, and the weight percentage of the catalysts (antimony(III) oxide and zinc acetate) varied between 0.025–1.0 wt %. The successful glycolysis reactions were conducted at 240 °C (Scheme 1).

**Figure 1:**
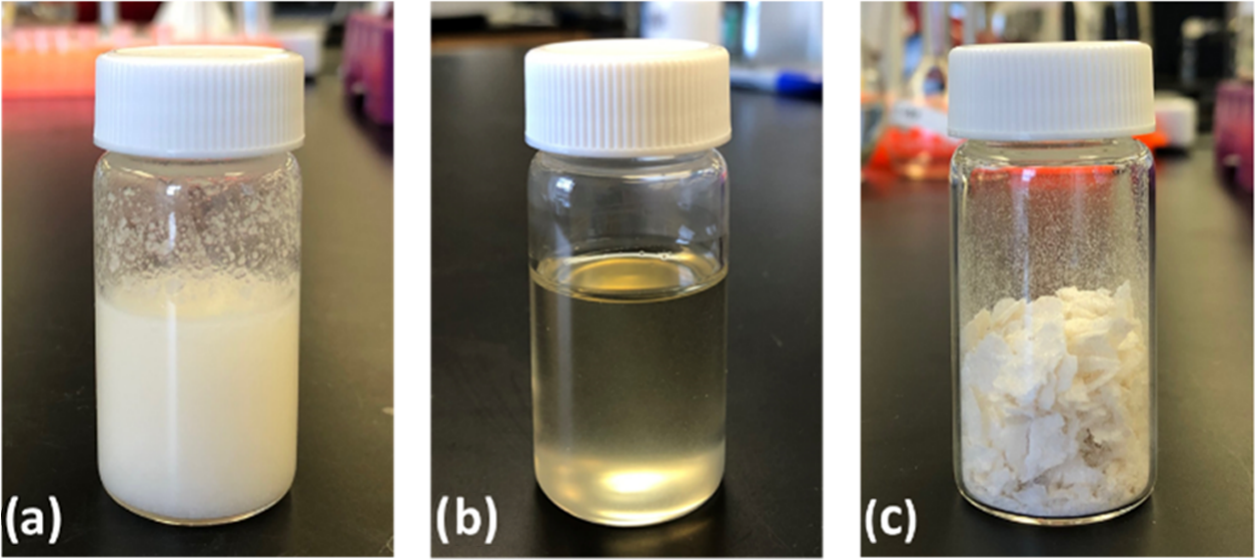
(a) White pasty reaction mixture in the presence of Sb_2_O_3_ at 70 °C, (b) transparent and one-phase reaction crude at 70 °C using zinc acetate, and (c) white crystals of BHET recovered from the reaction mixture after crystallization using zinc acetate.

The following procedure was used to conduct the PET glycolysis: the reaction mixture comprising PET waste, EG, and the catalyst were transferred to the EasyPrep Teflon vessel, which was then closed and placed into the microwave. The reaction mixture reached 240 °C in about 10 min (this is the shortest ramping time) and was kept at that temperature for the required period of time. The microwave irradiation was then set to turn off in order to cool down to 70 °C, in about 30 min. Before the reaction, the reaction mixture containing solid PET, solid catalyst, and liquid EG was heterogeneous, in contrast to the contents characterized after the reaction, recovered at 70 °C. The reaction mixture with the Sb_2_O_3_ catalyst was recovered as a white viscous paste (Figure 1a), but the reaction mixture in the presence of Zn(OAc)_2_ was recovered as a one-phase solution (Figure 1b). Afterward, a certain weight of the reaction mixture (around 50 mg) was taken for the HPLC measurements to calculate the yield of the BHET. The one-phase reaction mixture (Figure 1b) composed of BHET and possible PET oligomers in EG produced a white precipitate upon cooling to room temperature like the mixture in Figure 1a. A portion of the reaction mixture was vacuum filtered and vacuum-dried at 65 °C overnight to remove the remaining EG. This sample was used for FTIR, DSC, TGA, NMR, and viscometry analysis as the “crude reaction mixture”.

In order to purify the product, another portion of the Sb_2_O_3_-catalyzed reaction paste (Figure 1a) was purified by washing with water and dried in a vacuum oven at 65 °C to constant weight. The BHET-rich products catalyzed by zinc acetate was poured into cold deionized water (4 °C) and was kept at 4 °C in a refrigerator overnight for the white crystalline BHET to precipitate. Afterward, it was filtered and its product, BHET, was dried in a vacuum oven at 65 °C for 24 h and weighed (Figure 1c). The PET conversion was calculated based on eq 1, while the yield of BHET was measured using a HPLC calibration curve prepared with a solution of commercial BHET in methanol. The yield of oligomers was calculated based on eq 2. The yield was confirmed by weighing the nonsoluble oligomers in some oligomer-richer samples by washing with hot methanol, filtration, and drying and then comparing the weight against eq 2.
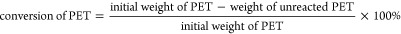
1
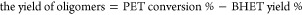
2

## 3 Results and Discussion

### 3.1 Optimization of the Reaction Conditions in the Presence of Antimony(III) Oxide

PET glycolysis reactions in the microwave have a higher reaction rate when compared to the conventional electrical heating process.^30^ To find the reaction conditions that lead to the formation of higher oligomers instead of formation of the BHET monomer, the effects of reaction time, reaction temperature, catalyst loading, and the EG/PET weight ratio were investigated. Table 1 shows the reaction conditions for the glycolysis of PET waste from water bottles, PET conversion, the BHET yield, and oligomer yield. A successful glycolysis method that produced the oligomer as the desired product in high yield in a reasonably short reaction time was done in the presence of 0.25% Sb_2_O_3_ wt % and a EG/PET weight ratio of 2.5 with 10 min ramping time to reach 240 °C with a 5 min holding time at 240 °C. We measured the reaction pressure at 240 °C for the PET glycolysis in a separate sealed reactor to be 2.5 bar.

**Table 1:** Screening of the Experimental Conditions of the Microwave-Assisted PET Glycolysis^a^

entry	catalyst (wt %)	ramping time (min)	holding time (min)	temperature (°C)	EG/PET (w/w)	conversion (%)	yield of BHET (%)	yield of oligomer *n* > 2 (%)
**M0**	no catalyst	10	5	240	5.0	30	1.0 ± 0.5	29.0
**M1**	Sb_2_O_3_ (0.50%)	10	25	200	2.5	0.0	0.0	0.0
**M2**	Sb_2_O_3_ (0.50%)	40	20	240	5.0	100	23.4 ± 0.5	76.6
**M3**	Sb_2_O_3_ (0.50%)	40	20	240	2.5	100	22.7 ± 0.3	77.3
**M4**	Sb_2_O_3_ (0.50%)	40	20	240	1.5	100	25.4 ± 0.1	74.6
**M5**	Sb_2_O_3_ (0.50%)	15	15	240	2.5	100	23.4 ± 0.1	76.6
**M6**	Sb_2_O_3_ (0.50%)	15	5	240	2.5	100	16.3 ± 0.03	83.7
**M7**	Sb_2_O_3_ (0.50%)	10	5	240	2.5	100	10.4 ± 0.06	89.6
**M8**	Sb_2_O_3_ (0.50%)	10	25	220	2.5	35	3.4 ± 0.01	31.6
**M9**	Sb_2_O_3_ (0.40%)	10	5	240	2.5	100	4.0 ± 0.01	96.0
**M10**	Sb_2_O_3_ (0.30%)	10	5	240	2.5	100	3.5 ± 0.01	96.5
**M11**	Sb_2_O_3_ (0.25%)	10	5	240	2.5	100	3.3 ± 0.01	96.7
**M12**	Sb_2_O_3_ (0.50%)	10	45	240	5.0	100	41.2 ± 0.7	58.8
**M13**	Sb_2_O_3_(0.50%)	10	35	240	5.0	100	46.3 ± 0.8	53.7
**M14**	Sb_2_O_3_(0.50%)	10	45	240	7.0	100	53.6 ± 0.5	46.4
**M15**	Zn (OAc)_2_ (0.25%)	10	5	240	5.0	100	80.1 ± 0.5	19.9
**M16**	Zn(OAc)_2_ (0.25%)	10	15	240	5.0	100	70.5 ± 0.2	29.5
**M17**	Zn(OAc)_2_ (0.25%)	10	25	240	5.0	100	54.8 ± 0.2	45.2
**M18**	Zn(OAc)_2_ (0.15%)	10	5	240	5.0	100	59.3 ± 0.8	40.7
**M19**	Zn(OAc)_2_ (0.15%)	10	25	240	5.0	100	64.8 ± 0.4	35.2
**M20**	Zn(OAc)_2_ (0.05%)	10	5	240	2.5	100	86.9 ± 0.4	13.1
**M21**	Zn(OAc)_2_ (0.04%)	10	5	240	2.5	100	96.3 ± 0.5	3.7
**M22**	Zn(OAc)_2_ (0.025%)	10	5	240	2.5	100	66.7 ± 0.6	33.3

a All 240 °C reactions were performed at 400 W powers.

Parameters such as type of the catalyst and its loading, temperature, ramping, and holding times, and EG to PET weight ratio can all have a significant impact on the outcome of the glycolysis reactions under microwave irradiation. Some of these conditions are chosen based on our previous results that showed the Sb_2_O_3_ as an effective catalyst for PET glycolysis,^29^ in which we used a high-pressure reactor. The choice of these factors is based on the following considerations. For the type of the catalyst and its loading, we chose Sb_2_O_3_ and the well-known Zn(OAc)_2_ in order to find the minimum loading of each catalyst that promotes complete PET conversion. The reaction temperature as well as the ramping and holding times are chosen and optimized to provide 100% PET conversion in the lowest possible temperature and time. Besides the 100% PET conversion, our main focus was on optimizing the yield of the oligomers, so the reaction conditions were adjusted to favor the formation of the oligomer while minimizing the formation of BHET in the presence of the catalyst.

### 3.2 Effect of EG/PET Weight Ratio

As shown in Table 1, the effect of EG/PET weight ratio (5.0 vs 2.5 vs 1.5) on the yield of BHET was investigated using 0.5 wt % loading of Sb_2_O_3_ as the catalyst, 40 min ramping, and 20 min holding time at 240 °C (samples M2, M3, and M4). The results demonstrated that a BHET yield of 23.4% was obtained in one hour (40 min ramping time, 20 min holding time, 240 °C) for the EG/PET weight ratio of 5.0, but it decreased slightly to 22.7% for the EG/PET weight ratio of 2.5 and increased to 25.4% on lowering the EG/PET weight ratio to 1.5. Therefore, based on these results, the EG/PET weight ratio of 2.5 was chosen for prioritizing the production of PET-glycolyzed oligomers.

### 3.3 Effect of Reaction Time and Temperature on the Glycolysis of PET

**Figure 2:**
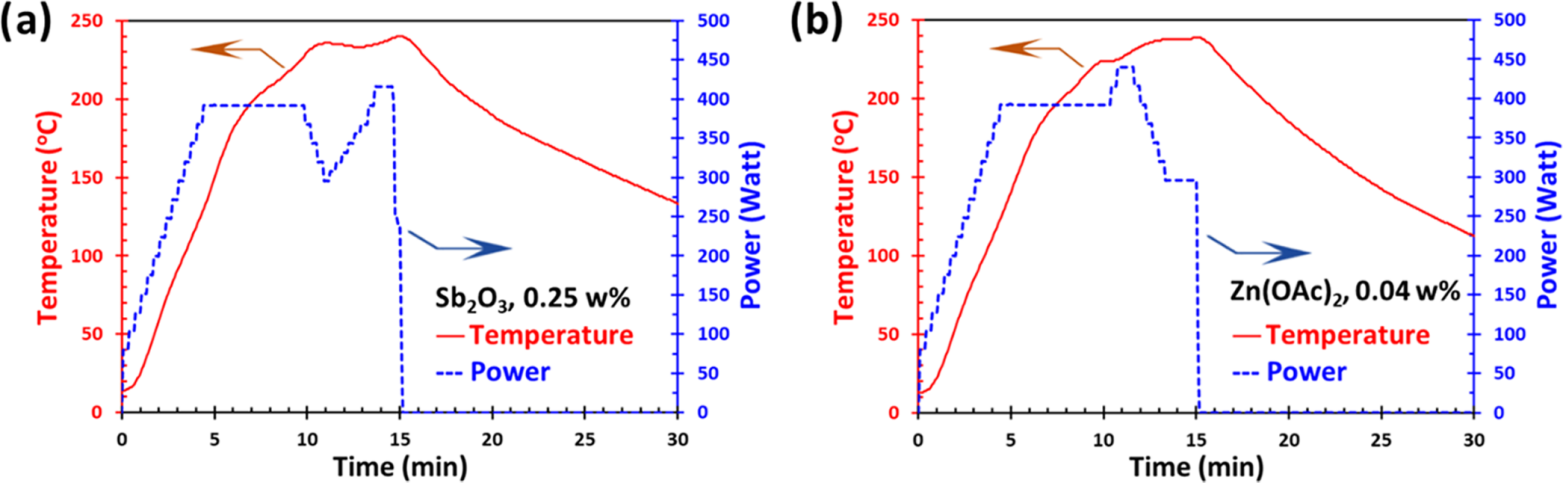
Temperature–time–microwave power profile for the sample M11 (Sb_2_O_3_, 0.25 wt % loading) (a) and sample M21 (Zn(OAc)_2_, 0.04 wt % loading) (b).

In order to investigate the effect of reaction time, the operation conditions for the reaction conditions were controlled to 240 °C and an EG/PET weight ratio of 2.5 with 0.50 wt % Sb_2_O_3_ loading. As Table 1 shows (samples M3, M5–M7), when the reaction time was 1 h (40 min ramping time, 20 min holding time), the yield of BHET was 22.7%, while when the ramping time (to achieve 240 °C) was decreased to 15 min, no noticeable change in yield of BHET was seen (23.4%), but by decreasing the holding time at 240 °C to 5 min, the yield of BHET was decreased to 16.3% (sample M6). As a result, the proper time for gaining the lowest level of monomer BHET and the highest level of oligomer is 10 min ramping time to 240 °C with 5 min holding time. According to Figure 2, the diagram of time, temperature, and power of the microwave through the reaction, the temperature of the reaction mixture reached 240 °C after 10 min with a set power of 400 W and after being held for 5 min, it started cooling to 70 °C over 30 min.

To determine the proper temperature during the reaction for PET glycolysis, we ran the reaction at 200, 220, and 240 °C with 0.50 wt % of Sb_2_O_3_ (10 min ramping time, 25 min holding time). It was shown that there was a significant decrease of conversion of PET from 100% to 0.0 for 200 °C and 35% for 220 °C as shown in Table 1 (samples M1 and M8). These results clearly showed that a temperature higher than 220 °C (and microwave power more than 300 W) is essential for the complete conversion of PET.

### 3.4 Evaluation of the Effect of the Amount and Type of Catalysts on PET Degradation

The PET glycolysis was done in the presence of different amounts of Sb_2_O_3_ catalyst to evaluate the effect of catalyst loading. In the absence of a catalyst (Table 1, sample M0), the yield of BHET was 1.0% with a PET conversion of 30%. The Sb_2_O_3_ loadings of 0.50, 0.40, 0.30, and 0.25 wt % were used to investigate the minimum required catalyst for a PET conversion of 100% while keeping the BHET yield as low as 10% (resulting in oligomer yields higher than 90%). The best results were found when 0.25 wt % of Sb_2_O_3_ was employed at 240 °C, with the EG/PET weight ratio of 2.5, leading to an oligomer yield of 96.7 and 100% PET conversion in 15 min (Table 1, sample M11). However, there is no significant difference between the 0.50 wt % catalyst loading and 0.40, 0.30, and 0.25 wt % (Table 1, samples M7, M9–M11). These results established that antimony(III) oxide would be a proper catalyst for PET oligomer synthesis using 0.25 wt % loading under microwave irradiation. Lower amounts of Sb_2_O_3_ resulted in PET conversions lower than 100% and therefore were not further studied.

In order to evaluate the effectiveness of the Sb_2_O_3_ in oligomer synthesis, we compared our results with the well-known catalyst for PET glycolysis, zinc acetate (Zn(OAc)_2_). Based on the BHET yields in Table 1, it was shown that zinc acetate exhibited very high catalytic activity for production of the BHET monomer under the microwave irradiation, even with a very low loading of 0.04 wt % (sample M21, 96.3% yield of BHET). In the best conditions that optimize the production of oligomers, the yield of oligomers can be only up to 45.2% (sample M17, 54.8% yield of BHET). As a general trend, it can be seen that the BHET yield decreases with the decrease of zinc acetate in the same conditions (compare M15 with M18 and M21 with M22). Interestingly, comparing the results of Sb_2_O_3_ and Zn(OAc)_2_, we can see that when the reaction was allowed to proceed under microwave irradiation, the choice of catalyst can determine the main product. By using Sb_2_O_3_, the PET conversion reaches 100% and oligomer formation reaches 97%, but in the presence of zinc acetate, the PET conversion reaches 100% whilst the BHET monomer formation reaches 96%.

The mechanism of the selectivity of antimony catalysts for oligomer formation is not yet fully understood, but it is believed to be due to the unique properties of antimony compounds, such as their high Lewis acidity and ability to stabilize the transition states of the reaction.^31^ On the other hand, Zn(OAc)_2_ is known as one of the best and fastest catalysts for PET glycolysis and has rarely been used for PET polymerization.^32^ When zinc acetate is used for polymerization of BHET to produce PET, even under strong vacuum to remove the EG at 260–293 °C, which is well above its boiling temperature, the degradation reaction competes with the polycondensation and decreases the molecular weight of the polymer.^32^ As a result, the molecular weight of the polymer reached 30,000 g mol^–1^ and further decreased over time. Some research groups^33^ investigated the mechanism and kinetics of the PET glycolysis using Zn(OAc)_2_. They propose a mechanism for the reaction that involves the formation of a zinc–PET intermediate that pushes forward the glycolysis reaction for producing BHET. Zinc acetate with excess EG favors the formation of the BHET monomer with a short reaction time and high selectivity and efficiency, while antimony oxide would favor polymerization of BHET under the right conditions, specifically high temperatures close to the polymerization temperature (260 °C).

### 3.5 Study of the Effect of Catalysts and Reaction Conditions on the Molecular Weight Distribution of PET Oligomers

We investigated how the reaction time and catalyst affect the depolymerization of PET by analyzing the molecular weight of the oligomers produced. To achieve this, we used dilute-solution viscometry by dissolving PET or oligomers in a solution of phenol/tetrachloroethane in 60:40 weight ratio to prepare a concentration of 0.5% (w/w). We then performed intrinsic viscosity measurement using an Ubbelohde size 1C viscometer at 25.0 °C by measuring the time required for the solution to pass through a fixed distance under gravity. By comparing this time to the time for a blank solution, we calculated the specific viscosity (η_sp_) and intrinsic viscosity [η] using eq 3 with a value of *K*′ equal to 0.35.^34^
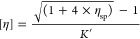
3

We then determined the molecular weight of PET and oligomers using the Mark–Kuhn–Houwink equation (eq 4) and the MKH parameters for PET, *K* and α, which were 2.1 × 10^–4^ and 0.82, respectively, as reported in the literature^34,35^
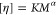
4

Table 2 shows the molecular weights (MWs) of the oligomers calculated using eq 4. The molecular weight of the PET sample from the waste water bottle used in the reaction was found to be 80,050 g mol^–1^, which corresponds to a degree of polymerization (DP) of 417. Our results showed a significant decrease in molecular weight of the glycolyzed fraction of the sample M0 after removing the unreacted PET, which is glycolysis without catalyst, from 80,050 to 13,210 g mol^–1^ (DP = 69). While sample M0 showed a 30% conversion, with the addition of antimony(III) oxide as the catalyst and on increasing the reaction time, the PET conversion increased to 100%, which means complete depolymerization. Sample M4, which is glycolysis using 0.50% Sb_2_O_3_ with 40 min ramping time and 20 min holding time at 240 °C, was characterized with a molecular weight of 3800 g mol^–1^ (DP = 20), while sample M7 using 0.50% Sb_2_O_3_ with 10 min ramping time and 5 min holding time at 240 °C indicated a molecular weight of 4450 g mol^–1^ (DP = 23). The increase in the average molecular weight of M7 compared to M4 is due to the shorter reaction time in M7 that causes a lower degree of ester bond cleavage as well as a higher oligomer yield in M7 compared to M4, which is 89.6 and 74.6%, respectively. When the amount of catalyst decreased from 0.50 to 0.25% followed by a shorter reaction time (10 min ramping time and 5 min holding time at 240 °C) in sample M11, we observed an increase in the molecular weight of oligomers to 7030 g mol^–1^ (DP = 37). This clearly shows that by reducing the catalyst loading and lowering the reaction time, while a 100% PET conversion can still be achieved, the molecular weight of the PET oligomers, i.e., the degree of ester bond cleavage, can be controlled. Furthermore, by using 0.50% Sb_2_O_3_ and increasing the time of the reaction and amount of EG in sample M12, the depolymerization rate increased, which led to a lower molecular weight of oligomers of 3890 g mol^–1^ (DP = 20). While the average molecular weight of the M12 sample is about the same as that of the M4 sample, the yield of oligomers in M12 is much lower compared to M4 (58.8 vs 74.6%) as the reaction proceeded to more ester bond cleavage to produce more BHET. As shown in Table 1, the Zn(OAc)_2_ catalyst tends to produce the BHET monomer instead of the PET oligomers, and the highest oligomer yield that we obtained is for sample M17 with 0.25% catalyst loading, a ramping time of 10 min, and a holding time of 15 min at 240 °C. The average molecular weight of oligomers in the M17 sample with an oligomer yield of 45.2% is 3960 g mol^–1^, which corresponds to a DP of 21. The molecular weight results shown in Table 2, combined with the HPLC results for the BHET yield from Table 1, show the tendency of Sb_2_O_3_ to produce oligomers, while Zn(OAc)_2_ is more effective toward complete cleavage of the ester bonds. This is most likely due to the fact that Sb_2_O_3_ is one of the most used catalysts for PET synthesis, which has high efficiency to polymerize BHET.^36,37^

**Table 2:** Viscosity and Molecular Weight of Some Samples

sample	intrinsic viscosity (dL/g)	molecular weight (g/mol)
PET waste	2.203	80,050
M0	0.503	13,210
M4	0.181	3800
M7	0.206	4450
M11	0.300	7030
M12	0.184	3890
M17	0.187	3960

### 3.6 Product Analysis

Different analytical techniques (NMR, DSC, TGA, HPLC, and IR) were used in order to characterize the reaction mixture and identify the structure of the main product(s) after PET waste glycolysis. These results were compared with the starting PET waste and a commercial BHET sample from Sigma-Aldrich.

#### 3.6.1 FTIR Analysis

**Figure 3:**
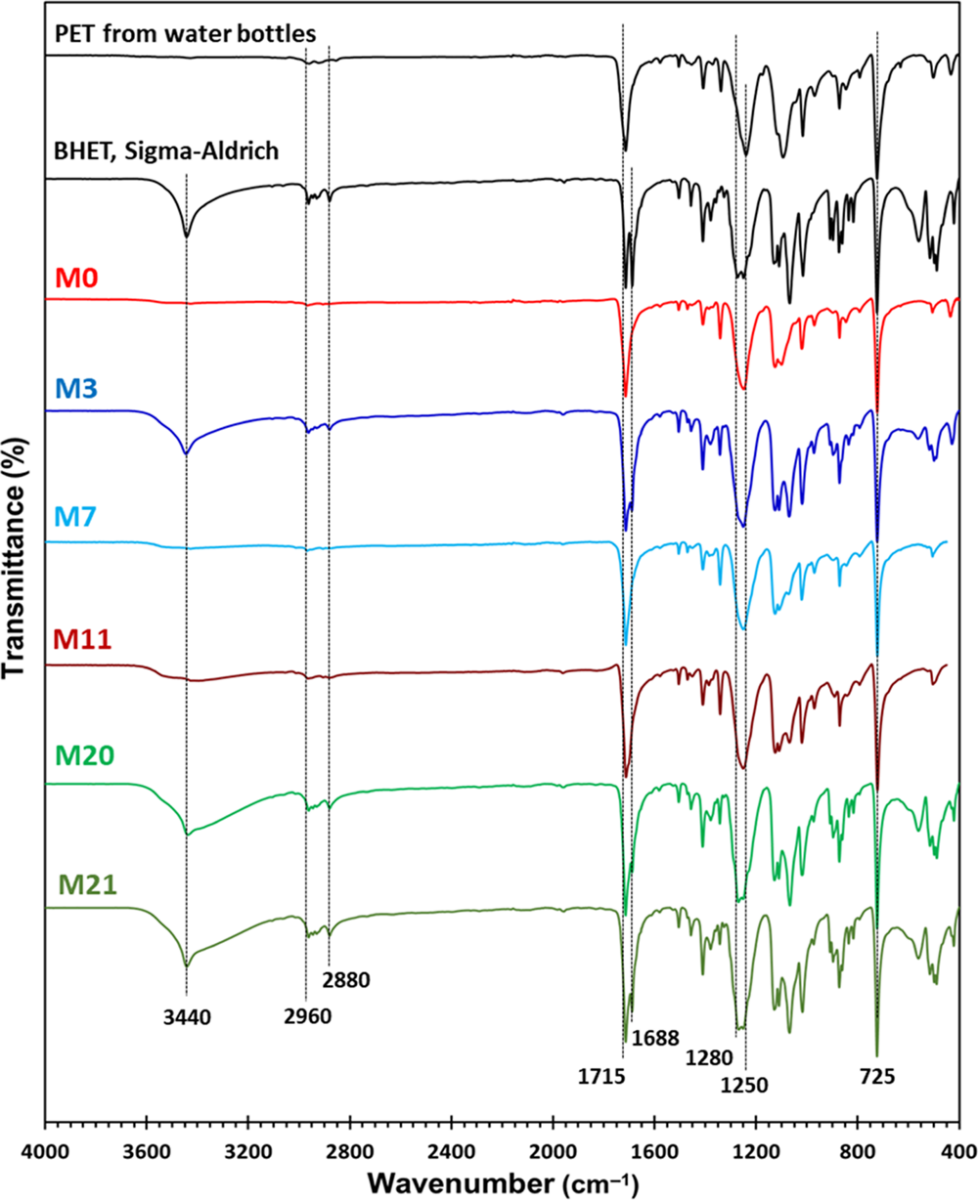
FTIR spectrum of PET waste from water bottle, BHET (Sigma-Aldrich), and crude reaction products obtained from PET glycolysis in reactions M0, M3, M7, M11, M20, and M21.

Figure 3 shows the FTIRs of PET (obtained from a water bottle) and pure BHET (Sigma-Aldrich), which were compared to the FTIR spectra of crude reaction mixtures from different runs including M0 (no catalyst), M3 (Sb_2_O_3_ 0.5 wt %, 60 min), M7 (Sb_2_O_3_ 0.5 wt %, 15 min), M11 (Sb_2_O_3_ 0.25 wt %, 15 min), M20 (Zn(OAc)_2_ 0.05 wt %, 15 min), and M21 (Zn(OAc)_2_ 0.04 wt %, 15 min). The spectrum of BHET showed two peaks at 3440 and 1130 cm^–1^, which are due to the OH group in BHET.^38,39^ As it can be seen in Figure 3, the low intensities of these peaks in M0, M7, and M11 clearly demonstrate the low BHET yield and high yield of oligomers in the presence of Sb_2_O_3_. In contrast, these peaks are sharp in M20 and M21, which show the high BHET yield in the presence of zinc acetate. In addition, the small peaks at 2960 and 2880 cm^–1^ indicate the alkyl C–H groups in BHET that again are weak in M1, M7, and M11, indicating the low density of BHET in contrast to the high density of BHET in M20 and M21. The absorption peaks at 1688 and 700–800 cm^–1^ indicate a benzene ring and a C=O stretching vibration appears at 1715 cm^–1^. The two peaks at 1250 and 1280 cm^–1^ demonstrate the vibration of the C–O–C bond. The medium-intensity peaks at 1457–1505 cm^–1^ correspond to the aromatic C–H groups in the BHET monomer.^38^ These results confirm the presence of BHET as the main product in the presence of zinc acetate and PET oligomers as the main product in the presence of antimony oxide as catalyst.

#### 3.6.2 NMR Analysis

**Figure 4:**
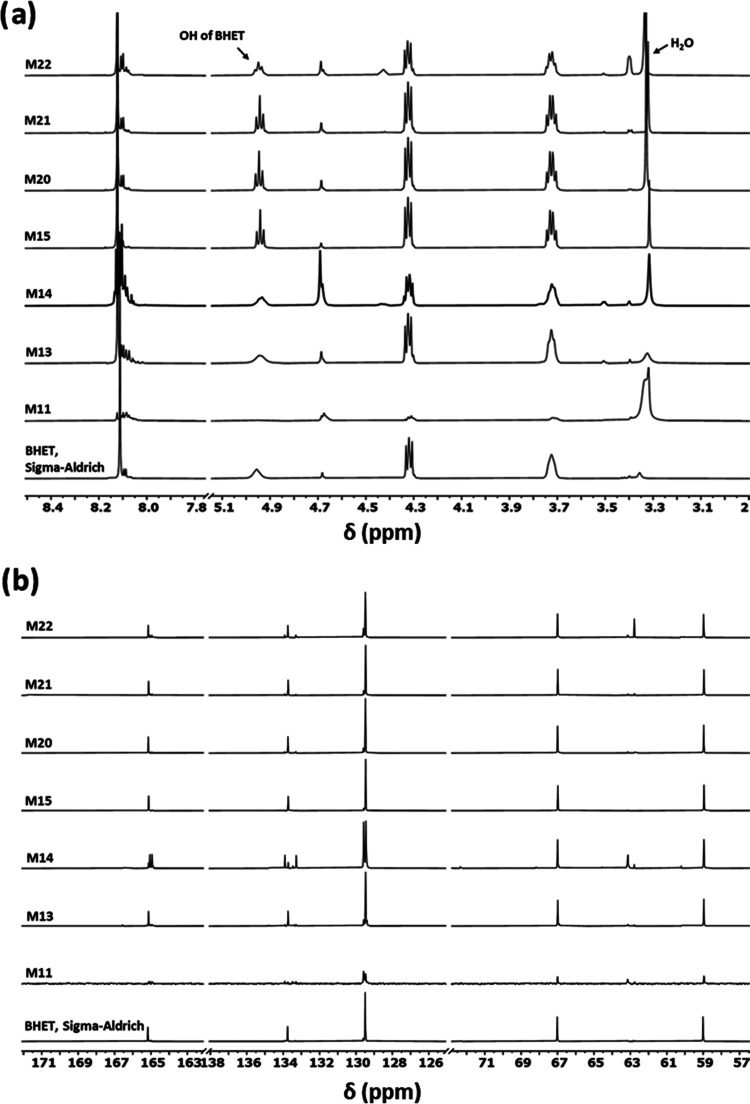
^1^H (a) and ^13^C NMR (b) spectra of BHET (Sigma-Aldrich) and some of the crude reaction mixtures from PET glycolysis reactions.

The ^1^H and ^13^C NMR spectra of the products from different runs have been studied. In the ^1^HNMR spectra (Figure 4), the peak at 8.12 ppm was characterized as the four aromatic protons of BHET and the signals at 4.32 and 3.72 ppm indicate the methylene protons of COO–CH_2_ and CH_2_–OH in BHET, respectively. The triplet at 4.92 ppm was characterized to the protons of hydroxyl in the BHET.^18,19^ As shown in Figure 4, the significant decrease in intensity of all these signals, especially the hydroxyl group at 4.29 ppm, shows the low yield of the BHET formed from the reaction M11 (3.3%). It is important to note that only BHET, and the dimer and trimer of PET show enough solubility in DMSO-*d*_6_ and that higher oligomers cannot be dissolved in this NMR solvent. A comparison of the spectra of M11, M13, and M14 samples demonstrates increase in the BHET formation (from 3.3 to 46.3 and 53.6%) with increasing reaction time in the presence of Sb_2_O_3_. It can be seen in the spectrum of the M14 sample (Sb_2_O_3_, 0.50%, 55 min) that with increasing time and on increasing the EG/PET weight ratio, the intensity of the small singlet at 4.69 ppm (Figure 4a), corresponding to the dimer of BHET,^17^ increased, demonstrating the polycondensation of BHET to a dimer with prolonged reaction time. The high intensities of all BHET monomer signals in M20, M21, and M22 indicate the high yield of BHET formation in the presence of zinc acetate as a catalyst.

The ^13^C NMR spectra of these samples (Figure 4b) are in accordance with their ^1^H NMR data. For BHET (Sigma-Aldrich), there are five peaks at 59.0, 67.0, 129.5, 133.8, and 165.2 ppm in the ^13^C NMR.^29^ The BHET dimer shows extra peaks at around 63, 130, 134, and 165 ppm. Since the solubility of PET oligomers is low in DMSO, especially for the higher oligomers (*n* > 2), none of the BHET and dimer signals can be observed in appreciative intensities in the spectrum of sample M11 (96.7% oligomers), which is a mixture of oligomers bigger than the dimer.

**Figure 5:**
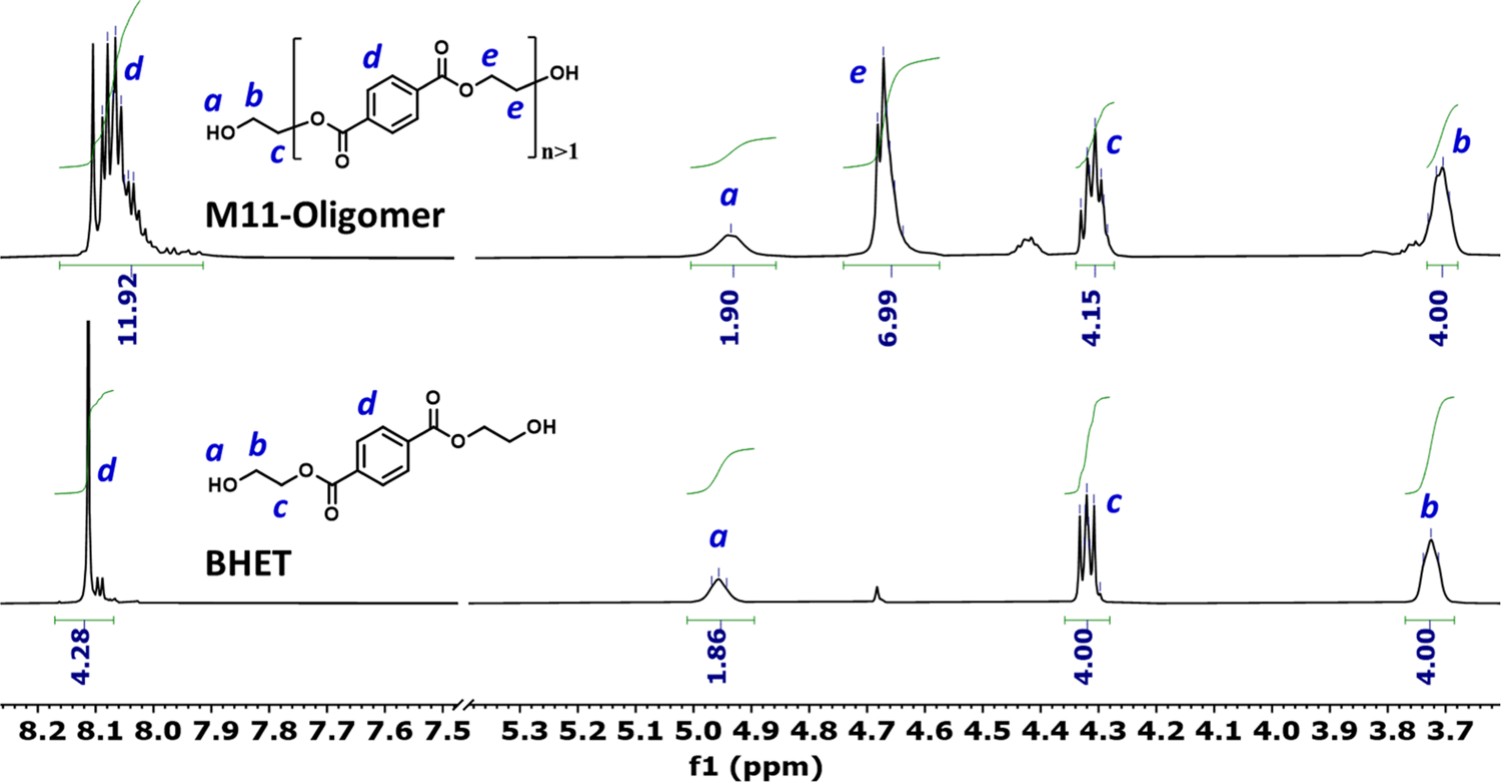
Comparison of the integrated areas of the methylene peak in the BHET (3.7 and 4.3 ppm) and methylene protons in oligomer (4.69 ppm).

The presence of oligomers can be confirmed by the detection of a methylene proton peak at 4.69 ppm, which belongs to the methylene groups of EG that reacted from both sides (internal EG units). As Figure 5 shows, the NMR spectrum of the BHET does not show this peak because it does not contain an internal EG unit. To determine the relative DP of oligomers using ^1^H NMR, the integration of the methylene proton signals in the oligomers (peak e at 4.69 ppm, Figure 5) should be used and compared with BHET’s methylene integral value (peak b vs peaks d and e). By comparing the integrated areas of the methylene and aromatic peaks in BHET (3.7, 4.3, and ∼8 ppm) with those peaks in the oligomers plus the 4.69 ppm peak, it was clear that a trimer existed in the oligomer from sample M11. Our viscometry results show a DP of 37 for this sample; the large difference is because only PET oligomers with a DP of 1, 2, and 3 (monomer, dimer, and trimer, respectively) are soluble in DMSO-*d*_6_, while the higher oligomers are only soluble in the phenol/tetrachloroethane solution mixture. It is important to mention that in the samples with high oligomer content prepared for the NMR analysis, there is always an insoluble part for the higher oligomers. However, in the samples with a high amount of BHET and lower amount of higher oligomers, like M20 and M21, the DMSO-*d*_6_ solution was transparent. The application of ^1^H NMR spectroscopy for analyzing BHET and PET oligomers, combined with the results from the molecular weight measurements by viscometry, provided valuable insights into the effectiveness of the antimony oxide catalysts for oligomer production and their relative proportions.

#### 3.6.3 DSC Characterization

**Figure 6:**
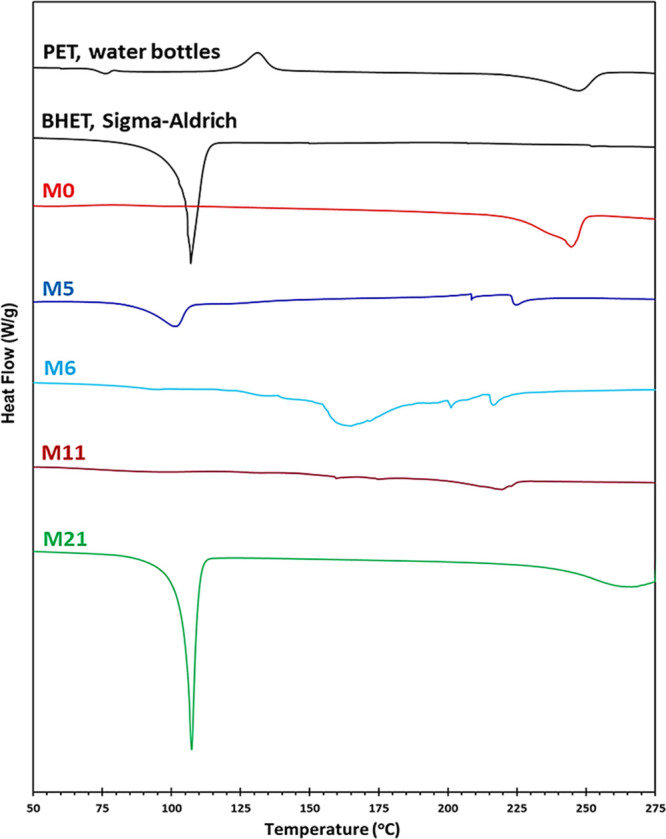
DSC thermograms of PET waste from water bottles, BHET (Sigma-Aldrich), and the main products obtained from PET glycolysis in different reaction conditions: M0, M5, M6, M11, and M21.

The DSC thermograms of BHET (Sigma-Aldrich), PET waste from water bottles, and some of the crude reaction mixtures from PET glycolysis are presented in Figure 6. BHET exhibits a sharp melting point at about 108 °C, which agrees with its known melting temperature.^40,41^ All samples from the reaction in the presence of zinc acetate, such as M21, exhibited endothermic peaks similar to the BHET monomer in the range of 100–115 °C for its melting. The M0 sample (PET glycolysis without any catalyst) indicated a broad peak at around 240 °C for the unreacted PET without showing a BHET peak. To show the effect of reaction time on the glycolysis product in the presence of 0.5 wt % Sb_2_O_3_, sample M5 (30 min reaction time) was compared to M6 (20 min reaction time). Sample M5 shows a small peak in the range of 100–115 °C, similar to the melting peak of BHET, but as a result of the decreased reaction time in sample M6, this peak was deleted and a broad peak at around 165 °C appeared, which is related to the oligomer of BHET. In sample M11, it can be seen that on decreasing the reaction time to 15 min (which comprises 10 min ramping time to 240 °C and 5 min holding time) and decreasing the catalyst loading to 0.25% Sb_2_O_3_, all peaks that were related to monomer BHET, the dimer, and the low-molecular-weight oligomer were deleted and only a broad peak appeared at around 220 °C, which belongs to the higher-molecular-weight oligomers of BHET as was shown by the viscometry analysis in Table 2.

#### 3.6.4 HPLC Analysis

**Figure 7:**
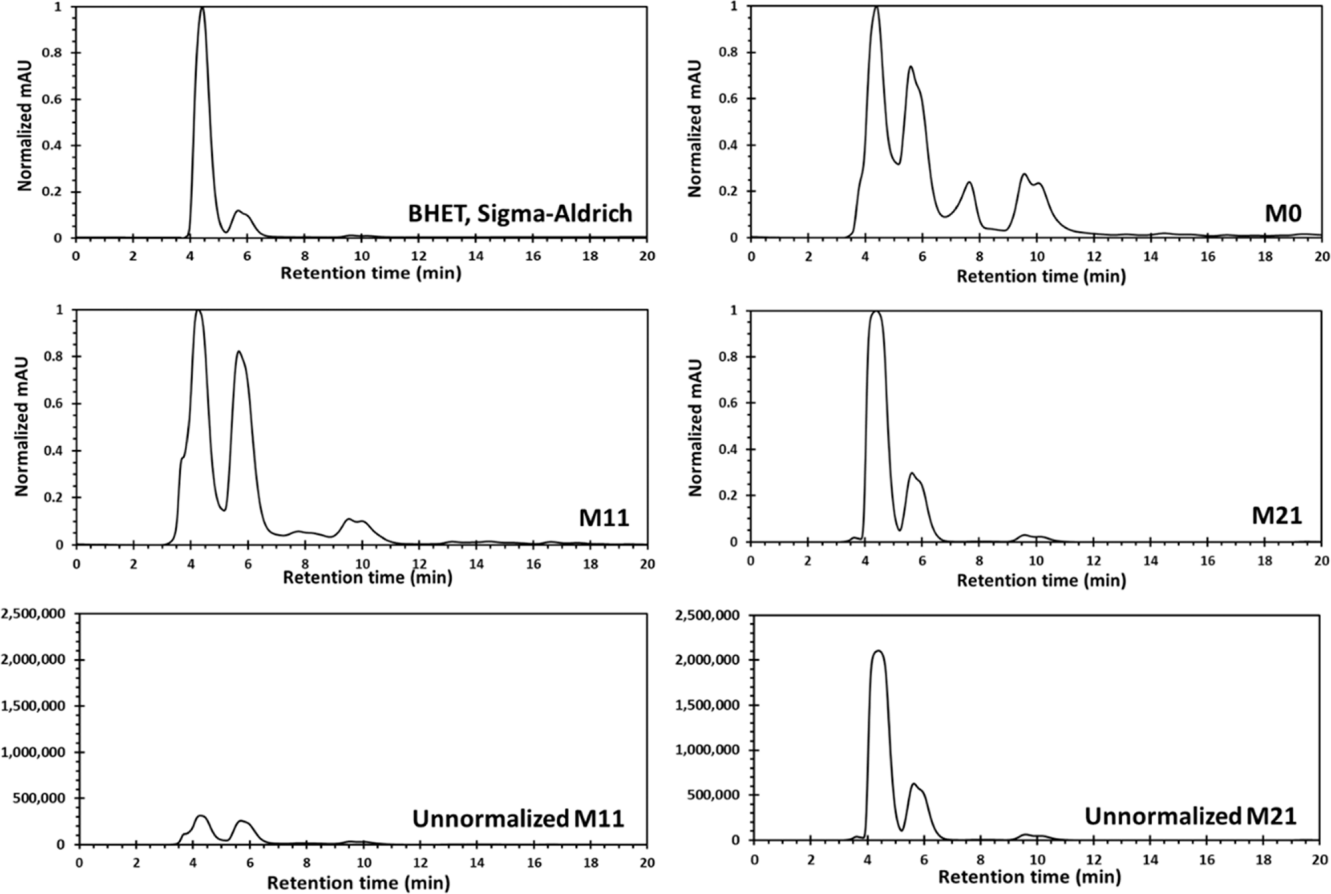
HPLC chromatogram of BHET (Sigma-Aldrich) and crude reaction mixture of PET glycolysis, from M0, M11, M21, and BHET. The intensities for the top four chromatograms are normalized for comparison purpose.

HPLC was used to identify and quantify the BHET monomer in the product from PET glycolysis using BHET from Sigma-Aldrich as a standard. The sample solution was prepared by dissolving about 50 mg of the crude glycolysis mixture in 10 mL of methanol, and the concentration of BHET was obtained using a calibration curve. As Figure 7 shows, the HPLC chromatogram of BHET shows one sharp peak at the retention time of 4.41 min, which shows the BHET monomer, and a small peak at the retention time of about 5.82 min, which corresponds to a small amount of BHET dimer. The chromatogram of some selected samples (M0, M11, and M21) is shown in Figure 7. The chromatogram of reaction M0 (without catalyst) shows different impurities beside the BHET (only 1% yield), which are the dimer and other lower oligomers of PET that are soluble in methanol at retention times of about 5.62, 7.65, 9.55, and 10.17 min. The chromatogram of M11 (Sb_2_O_3_, 0.25%, 15 min reaction time, 3.3% yield of BHET) shows very small peaks related to BHET, the dimer, and some oligomer peaks (see the unnormalized chromatogram in Figure 7). In comparison to M11, the chromatogram of reaction M21 (Zn(OAc)_2_ 0.04%, 15 min reaction time) shows one sharp peak at the retention time of 4.41 min, which belongs to the BHET monomer, and a small peak at the retention time of about 5.75 min, which corresponds to a small amount of the BHET dimer. In Figure 7, the normalized and unnormalized intensity chromatograms of samples M11 and M21 are shown for comparison purpose.

#### 3.6.5 TGA Study

**Figure 8:**
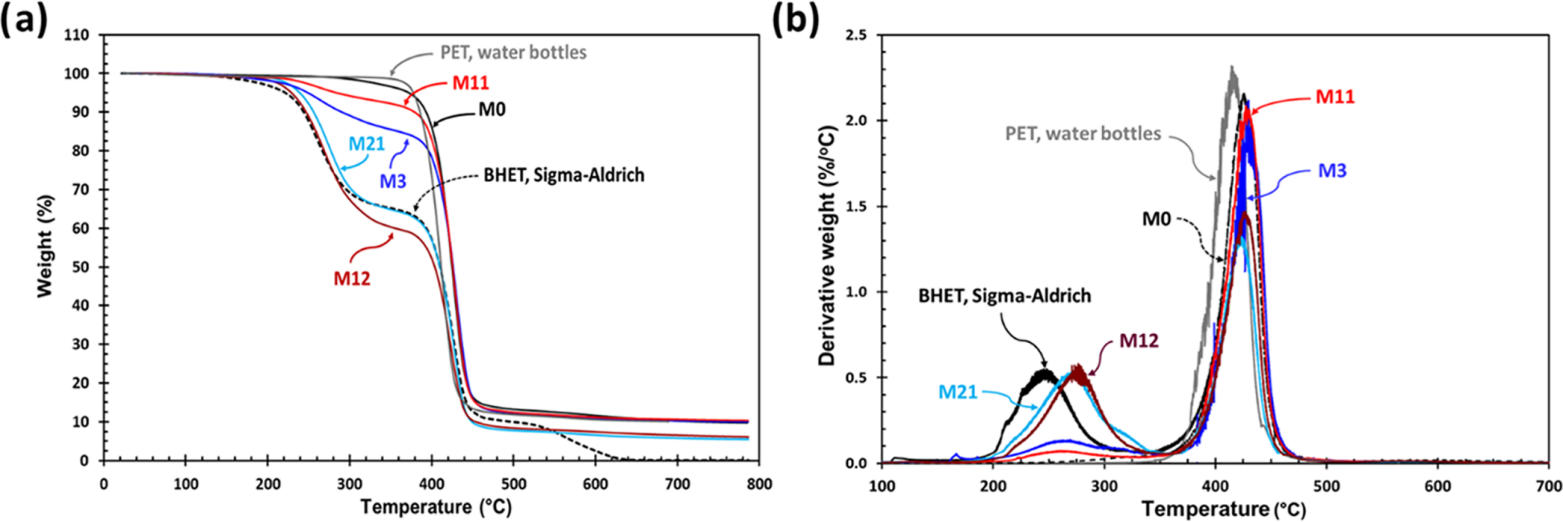
TGA thermograms (a) and its derivatives (b) of PET waste (water bottles), BHET (Sigma-Aldrich), and main PET glycolysis products from M0, M3, M11, M12, and M21.

TGA characterization can be useful for the study of the PET glycolysis reaction crude and its composition. TGA thermograms of PET waste, BHET, and some glycolyzed samples in the range of 25–800 °C were investigated to further analyze the products (Figure 8). The TGA thermogram of BHET indicates three different weight loss temperatures. The first weight loss in the temperature range 210–270 °C, at about 34%, is shown because of the polymerization of BHET and release of ethylene glycol; the second weight loss of around 52.2% is shown at 390–430 °C due to decomposition of PET oligomers that are produced through the heating of BHET; and the third decomposition occurred at 480–540 °C with a weight loss of around 11.5% because of the decomposition of the remaining BHET and PET that is formed during the heating of BHET in the TGA study.^42^Figure 8 compares the TGAs of different glycolysis samples with BHET and PET. The TGA thermograms of samples M0 show the presence of unreacted PET and other possible oligomers^43^ in the temperature range of 400–440 °C, with the weight loss (90.2%) indicating the lowest level of glycolysis occurring without a catalyst. The crude reaction mixture of sample M3 displays two weight loss intervals: the first one around 15.6% in the temperature range 230–260 °C, which indicates the thermal behavior of BHET, and the second one around 71.7% in the temperature range 400–440 °C, which is attributed to the thermal polymerization of BHET and also higher oligomers during the TGA processes. In comparison, the product from reaction M11 also showed similar peaks, 7.5% weight loss in the temperature range 230–250 °C, which shows the lowest level of BHET production, and 78.6% weight loss in the temperature range 400–440 °C, which can be attributed to the production of higher oligomers during the PET glycolysis reaction in the optimized reaction condition. The TGA thermogram of reaction M12 (increasing reaction time) demonstrates two major weight losses (39.2 and 51.2%), which showed a higher BHET and lower high-molecular-weight oligomer production in the presence of antimony oxide as a catalyst on increasing the reaction time. The TGA thermogram of reaction M21 (0.04% Zn(OAc)_2_) shows three weight losses (31.8, 59.1, 6.61%), which were the same as the thermogram data of BHET from Sigma-Aldrich.

## 4 Conclusions

We used antimony(III) oxide as a catalyst under microwave irradiation for PET waste glycolysis to produce only PET oligomers and not the ultimate degradation product, the BHET monomer. PET oligomers have potential uses in different applications; their isolation and purification is easier compared to the BHET monomer, all while consuming less energy and time in their production. Our results show that controlling the depolymerization of PET to oligomers is possible with 0.25 wt % antimony(III) oxide with the EG to PET weight ratio of 2.5 under microwave irradiation. In only 5 min at 240 °C and 400 W, up to 96.7% of PET oligomers with a DP of 37 was produced with only 3.3% BHET. We compared the results for antimony(III) oxide used as catalyst with those for zinc acetate, which is a well-known catalyst for PET glycolysis. At the same reaction conditions, under microwave irradiation, 0.04% zinc acetate produces almost entirely BHET with 96.3% yield. The fact that antimony(III) oxide is one of the best and widely used catalysts for PET industrial synthesis^37,44^ while zinc acetate is not is probably the reason for the differences that we observed for the activities of these two catalysts for PET glycolysis under microwave irradiation. Unlike zinc acetate, antimony(III) oxide has the tendency to polymerize the formed BHET monomer in the right conditions, as this is already one of the common catalysts for the industrial production of PET.^37^ The PET oligomers are synthesized with lower catalyst loadings, lower EG/PET weight ratio, and with shorter reaction time; hence, a lower energy/cost needed for their production. While the toxicity of Sb_2_O_3_ and its adverse health effects are well known,^45,46^ it should be noted that the main purpose of the current study is to show that the same catalyst that accounts for about 90% of PET manufacturing^45^ can be used for its partial depolymerization. We hypothesize that using these partially depolymerized oligomers with the same catalyst that was used for their production, Sb_2_O_3_, in the same reactor can be a cost-effective methodology for the synthesis of repolymerized PET from PET waste.
